# Aspects and Implementation of Pharmaceutical Quality by Design

**DOI:** 10.3390/pharmaceutics16060832

**Published:** 2024-06-19

**Authors:** Tamás Sovány, Katalin Kristó, Ildikó Csóka

**Affiliations:** Institute of Pharmaceutical Technology and Regulatoy Affairs, University of Szeged, Eötvös u. 6., H-6720 Szeged, Hungary; kristo.katalin@szte.hu (K.K.); csoka.ildiko@szte.hu (I.C.)

The introduction of the Quality by Design concept in 2004 has brought a paradigm shift in the pharmaceutical industry as well as a new era in pharmaceutical research and development [1]. This concept offers higher flexibility in production while reducing manufacturing costs [2] through the application of a complex risk-based approach from the beginning of the early development phase until the end of the product lifecycle.

The Quality by Design concept is a highly multidisciplinary field, as the implementation of the many aspects of this complex approach requires the use of versatile methodology and a wide range of tools, from understanding the patients’ needs [3], through the proper design of experiments [4,5], to advanced process analytical technologies [6,7], multivariate data analysis, and machine learning [8]. The papers published in this Special Issue well represent the broad applicability of this concept.

Sadamoto et al. focused on the fulfillment of patient needs with the development of a child-resistant but senior-friendly packaging (contribution 1). Their paper discusses the applicability of and cost-effectiveness of different materials that could be used for the manufacturing of press-through packages with the aim of creating packaging that is completely child-resistant but still applicable for older patients with rheumatoid arthritis or another strength impairment.

Sun et al. provided an analytic-focused paper that discusses the applicability of Raman mapping for reverse engineering purposes in generic drug development (contribution 2), which may offer a new way to better understand the structure and release mechanism of the original formula, reduce development time, and increase the success of product development.

Other papers in this Special Issue have focused on process optimization; for example, Lück et al. (contribution 3) critically evaluated the Johanson model and the derived Midoux number used for the mathematical prediction of ribbon solid fraction during roll compaction and investigated the effect of the roll speed on their prediction accuracy. They concluded that the roll speed exerts a significant influence on the obtained results and should be included in future models to increase the accuracy of predictions.

In addition to the application of mechanistic mathematical models, the most commonly used method in process optimization is the application of the design of experiments and response surface methodology (RSM). Alrobain et al. used it effectively for the improvement of tablettability of a poorly compactable and high-drug loading composition by optimizing the amount and mixing time of a nanosized colloidal silica excipient (contribution 4). Nevertheless, the versatility of this methodology is well demonstrated by the fact that the same research group effectively also utilized it in the optimization of the composition of sildenafil containing orodispersible minitablets (contribution 5) and for the determination of the design space of a green fluidized bed granulation process, which aimed to decrease water and energy consumption while keeping the optimized granule properties (contribution 6). The same methodology was employed by Németh et al. for the optimization of the zeta potential of liposomes (contribution 7) and by Frankiewicz and Szintowska for the optimization of a film coating process (contribution 8). Nevertheless, this latter research well demonstrated that the RSM may require many experiments, especially if the process parameters should be tested for multiple formulations. Nevertheless, the study of Hassan et al. (contribution 9), and Žiberna and Grabnar (contribution 10), which aimed to optimize a plain buccal mucoadhesive film, which was later successfully applied for the delivery of various drugs, and the development of olanzapine containing oral lyophilizates, respectively, showed that the proper evaluation and risk assessment of the existing knowledge space can effectively help in the determination of the most important factors and decrease the required number of experiments.

However, the study by Pielenhofer et al. (contribution 11) displays well that although the utilization of a proper risk assessment may effectively decrease the size of the experimental plan, the final number of experiments may still be high if the aim is not the better understanding of a certain production step but the development of an investigational product, whose production might consist of multiple consecutive manufacturing steps. The paper by Šahinović et al. (contribution 12) also faced similar problems and utilized an iterative optimization process to resolve them and decrease the final number of experiments.

Finally, the paper by Romero-Obon et al. (contribution 13) describes a new methodology for using retrospective data to narrow the number and range of variables.

The Guest Editors express their gratitude for the cooperation of the contributors, who are reputable researchers in this field and, with their published papers, helped to reveal the different aspects, provide a better understanding of the concept of pharmaceutical quality by design, and provide useful tools and methodology for conducting further research in this field.
